# Dissecting the polygenic contribution of attention-deficit/hyperactivity disorder and autism spectrum disorder on school performance by their relationship with educational attainment

**DOI:** 10.1038/s41380-024-02582-w

**Published:** 2024-05-23

**Authors:** Judit Cabana-Domínguez, Rosa Bosch, María Soler Artigas, Silvia Alemany, Natalia Llonga, Laura Vilar-Ribó, Pau Carabí-Gassol, Lorena Arribas, Valeria Macias-Chimborazo, Gemma Español-Martín, Clara del Castillo, Laura Martínez, Mireia Pagerols, Èlia Pagespetit, Raquel Prat, Julia Puigbó, Josep Antoni Ramos-Quiroga, Miquel Casas, Marta Ribasés

**Affiliations:** 1 Psychiatric Genetics Unit, Group of Psychiatry, Mental Health and Addiction, Vall d’Hebron Research Institute (VHIR), Universitat Autònoma de Barcelona, Barcelona, Spain; 2 Department of Mental Health, Hospital Universitari Vall d’Hebron, Barcelona, Spain; 3 Biomedical Network Research Centre on Mental Health (CIBERSAM), Madrid, Spain; 4 SJD MIND Schools Program, Hospital Sant Joan de Déu, Institut de Recerca Sant Joan de Déu, Esplugues de Llobregat, Spain; 5 Department of Genetics, Microbiology, and Statistics, Faculty of Biology, Universitat de Barcelona (UB), Barcelona, Spain; 6 Department of Psychiatry and Forensic Medicine, Universitat Autònoma de Barcelona (UAB), Barcelona, Spain; 7 Department of Clinical Foundations, Faculty of Medicine and Health Sciences, Universitat de Barcelona (UB), Barcelona, Spain; 8 Department of Medicine, Faculty of Medicine, Universitat de Vic-Universitat Central de Catalunya (UVic-UCC), Vic, Spain; 9 Sport and Physical Activity Research Group, Mental Health and Social Innovation Research Group, Centre for Health and Social Care Research (CEES), Universitat de Vic-Universitat Central de Catalunya (UVic-UCC), Vic, Spain; 10 Fundació Privada d’Investigació Sant Pau (FISP), Barcelona, Spain

**Keywords:** ADHD, Autism spectrum disorders, Genetics

## Abstract

Attention-deficit/hyperactivity disorder (ADHD) and autism spectrum disorders (ASD) are strongly associated with educational attainment (EA), but little is known about their genetic relationship with school performance and whether these links are explained, in part, by the genetic liability of EA. Here, we aim to dissect the polygenic contribution of ADHD and ASD to school performance, early manifestation of psychopathology and other psychiatric disorders and related traits by their relationship with EA. To do so, we tested the association of polygenic scores for EA, ADHD and ASD with school performance, assessed whether the contribution of the genetic liability of ADHD and ASD to school performance is influenced by the genetic liability of EA, and evaluated the role of EA in the genetic overlap between ADHD and ASD with early manifestation of psychopathology and other psychiatric disorders and related traits in a sample of 4,278 school-age children. The genetic liability for ADHD and ASD dissected by their relationship with EA show differences in their association with school performance and early manifestation of psychopathology, partly mediated by ADHD and ASD symptoms. Genetic variation with concordant effects in ASD and EA contributes to better school performance, while the genetic variation with discordant effects in ADHD or ASD and EA is associated with poor school performance and higher rates of emotional and behavioral problems. Our results strongly support the usage of the genetic load for EA to dissect the genetic and phenotypic heterogeneity of ADHD and ASD, which could help to fill the gap of knowledge of mechanisms underlying educational outcomes.

## Introduction

Attention-deficit/hyperactivity disorder (ADHD) and autism spectrum disorder (ASD) are complex childhood-onset neurodevelopmental disorders that often co-occur, are highly polygenic and share common genetic architecture [1]. Both conditions are associated with educational outcomes including educational attainment (EA) [2, 3], which also has substantial polygenic contribution and is linked to a broad range of outcomes [2, 4–10].

Although the genetic relationship between ADHD and ASD with EA has been widely studied, its complex nature still remains largely unclear. A large proportion of ADHD and ASD risk variants are shared with EA, but while almost all ADHD risk loci are associated with worse educational outcomes, ASD genetic variants showed mixed direction of effects [3, 11]. Consistently, there is a strong negative genetic correlation between ADHD and EA and a moderate positive genetic correlation between ASD and EA [1]. Recent findings support the existence of a polygenic form of pleiotropy that contributes to the positive genetic correlation between ADHD and ASD, and is consistent with the discordant polygenic association between these neurodevelopmental disorders and EA [12]. Also, polygenic scores for ADHD are associated with lower levels of EA, while polygenic risk for ASD is associated with higher cognitive functionality and EA [3, 13]. These distinct patterns of association with EA align with mendelian randomization results showing causal effects with opposite direction between these neurodevelopmental disorders and EA [14].

**Fig. 1:**
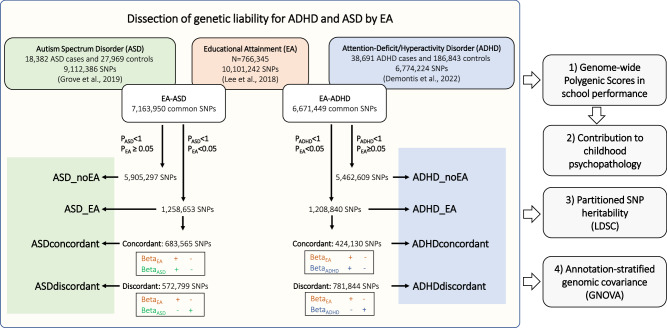
Study design. GWAS summary statistics on educational attainment (EA) (orange rectangle) [4], attention-deficit/hyperactivity disorder (ADHD) (blue rectangle) [3] and autism spectrum disorder (ASD) (green rectangle) [13] were used. The genetic liability of ADHD and ASD were dissected by their relationship with EA (see material and methods). With these genomic partitions we conducted genome-wide polygenic scores (PGS) analyses and test their association with school performance and early manifestation of psychopathology in our deeply phenotyped cohort of 4278 school-age children and adolescents and performed analyses of partitioned heritability and annotation-based stratified genetic covariance. “+” and “−” in the figure refer to the direction of the effect of the alleles studied.

Phenotypic associations between school performance, considered an early life intermediate phenotype that may anticipate EA [6–8, 15–17], and ADHD or ASD align with their pattern of genetic correlation with EA [18, 19]. Children with ADHD are at increased risk of learning disabilities and communication disorders, obtain lower grades than their peers without ADHD and are at greater risk of failure to graduate from high school on time [20–22], while academic functioning and achievement in ASD is highly variable [23–28]. However, little is known about the genetic underpinnings for the relationship between these neurodevelopmental disorders and school performance and whether these links are explained, in part, by the genetic liability of EA. We aim to dissect the polygenic contribution of ADHD and ASD to school performance by its relationship with EA in a deeply phenotyped cohort of 4278 school-age children and adolescents (see the workflow in Fig. 1) by (i) testing the association of polygenic scores for EA, ADHD and ASD with school performance, (ii) assessing whether the contribution of the genetic liability of ADHD and ASD to school performance is influenced by the genetic liability of EA and (iii) evaluating the role of EA in the genetic overlap between ADHD and ASD with early manifestation of psychopathology and other psychiatric disorders and related traits.

## Materials and methods

### Study sample

The study sample included 4278 school-age children and adolescents from the INSchool cohort (mean age = 10.03 years, SD = 2.95; 43.7% females) from 45 primary and secondary schools across Catalunya, Spain, with GWAS and data on school performance available. Inclusion criteria for the participants were European ancestry and written informed consent from parents/caregivers prior to participation. The project was approved by the Ethics Committee at the Hospital Universitari Vall d’Hebron and the Hospital maternoinfantil Sant Joan de Déu.

### School performance

School performance data were provided by schools and assessed with school grades in three different subjects: mathematics, foreign language and primary language. School grades were on a four-point scale, being A: excellent performance, B: good performance, C: adequate performance and D: underperformance.

### Clinical assessment

Childhood psychopathology was available for most of the school-aged sample (97%; *n* = 4135). Parents or surrogates completed the Child Behavior Checklist for ages 6–18 (CBCL-6/18) from Achenbach System of Empirically Based Assessment (ASEBA). ADHD symptoms were assessed with the Conners’ Rating Scale for parents (CPRS) [29]. Since no specific instrument assessing ASD symptoms was available in INSchool, we assessed ASD symptoms using a combination of 10 items from the Thought Problems, Attention Problems, Social Problems and the Withdrawn/Depressed scales of the CBCL-6/18, to generate an ASD-CBCL scale as previously described [30]. Details on the clinical evaluation can be found in Español-Martín et al. [31].

### Genotyping, imputation and quality control

Genomic DNA was isolated from saliva samples or buccal swabs collected using Oragene DNA OG-500 or OC-175 kits, respectively (DNA Genotek) and genotyped in three different genotyping waves with the Illumina Infinium PsychChip_v1.0 array for wave 1 (*n* = 794) or the Infinium Global Screening Array-24 version_2 (GSA_v2) for waves 2 (*n* = 2735) and 3 (*n* = 749) (Illumina, CA, San Diego, USA). Pre-imputation quality control was done with the PLINK 2.0 [32] and included individual and variant filtering based on the following parameters: variant call rate >0.95 (before individual filtering), individual call rate >0.98, autosomal heterozygosity deviation (|Fhet| < 0.2), variant call rate >0.98 (after individual filtering), SNP Hardy-Weinberg equilibrium (HWE; *P* > 1e−10) and minor allele frequency (MAF) > 0.01. Genetic outliers were identified by principal component analysis (PCA) using PLINK_2.0 and the mixed ancestry 1000G reference panel [33]. Ancestry outliers were excluded if their principal component (PC) values for PC1 or PC2 were greater than 1 standard deviation from the mean-centering point for our sample, considering each GWAS wave separately. Related and duplicated samples were identified by the “KING-robust kinship estimator” analysis in PLINK_2.0 [34] and one individual was excluded from each pair of subjects with kinship coefficient >0.0442. Imputation was done with McCarthy tools, for data preparation, and the Michigan Imputation Server [35], using the Haplotype Reference Consortium (HRC_Version_r1.1_2016) reference panel (GRCh37/hg19). Post-imputation dosage files with imputation INFO score >0.8 and MAF > 0.01 were considered for subsequent analyses.

### Genome-wide polygenic scores

Genome-wide polygenic scores (PGS) were constructed in our in-house sample from INSchool considering each genotyping wave separately using PRS-CS [36], PLINK_2.0 and summary statistics from the largest available GWAS to date on EA (*N* = 766,345 individuals) [4], ADHD (*N* = 38,691 cases and 186,843 controls) [3] and ASD (*N* = 18,382 cases and 27,969 controls) [13]. All PGS were computed and standardized to a mean of 0 and a standard deviation of 1.

PGS for ADHD or ASD were constructed with five subsets of SNPs based on their contribution to EA: (i) The overall set of SNPs from Demontis et al. (PGS_ADHD_; *n* = 6,774,224 SNPs) or Grove et al. (PGS_ASD_; *n* = 9,112,386 SNPs) [3, 13]; (ii) variants not associated with EA (PGS_ADHD_noEA_ and PGS_ASD_noEA_; *P*_EA_ > 0.05); (iii) variants associated with EA (PGS_ADHD_EA_ and PGS_ASD_EA_; *P*_EA_ < = 0.05); (iv) variants associated with EA and showing consistent direction of the effect in EA and ADHD (PGS_ADHDconcordant_; Beta_ADHD_ > 0 and Beta_EA_ > 0 or Beta_ADHD_ < 0 and Beta_EA_ < 0) or in EA and ASD (PGS_ASDconcordant_; Beta_ASD_ > 0 and Beta_EA_ > 0 or Beta_ASD_ < 0 and Beta_EA_ < 0), and (v) variants associated with EA and showing opposite direction of the effect in EA and ADHD (PGS_ADHDdiscordant_; Beta_ADHD_ > 0 and Beta_EA_ < 0 or Beta_ADHD_ < 0 and Beta_EA_ > 0) or in EA and ASD (PGS_ASDdiscordant_; Beta_ASD_ > 0 and Beta_EA_ < 0 or Beta_ASD_ < 0 and Beta_EA_ > 0) (Fig. 1).

In addition, PGS constructed on the subset of SNPs shared between ADHD_discordant_ and ASD_concordant_ partitions (*n* = 369,860 SNPs) were calculated using ADHD (PGS_ADHDshared_) and ASD (PGS_ASDshared_) weights separately.

### Genome-wide polygenic score analyses stratified by sex

The same methods described for all individuals were used for the sex stratified analyses, except for the inclusion of school as a random effect in the models, which was not possible due to the reduction in sample size. An interaction test using the Wald-type test implemented in the metafor R Package was performed to test differences observed between sexes. In addition, results from the analyses stratified by sex where there was a significant effect in boys but not in girls were confirmed using a permutation-based approach to rule out the effect of a difference in sample sizes between boys and girls (*n* = 2409 boys and 1869 girls). A bootstrap resampling (1000 permutations) was used to select subsets of boys with the same sample size as the girls, and the association analyses were repeated for these subsets.

### Statistical analysis

#### Genome-wide polygenic scores

The association between PGS and school performance was assessed with ordinal mixed-effect models considering D as the lowest and A as the highest category using the ordinal R package. In this context the effect estimate of the PGS produced by this model is an odds ratio, which provides the odds of an increase in school performance category per one PGS standard deviation. For instance, an odds ratio of 1.5 for a PGS in a specific subject performance would indicate that for an increase of one standard deviation in the PGS the odds of having a higher score in the specific subject is of 1.5. The percentage of variance attributable to each PGS was reported as the mean percentage of variance of each genotyping wave calculated as the increase in Nagelkerke’s pseudo-R2 between models with and without the PGS. For quantile plots, the target sample from each genotyping wave was divided into five quintiles of increasing PGS and PGS were compared across ranked quintiles considering the lower quintile as reference. To visualize how PGS affect school performance, predicted probabilities of being in each category (A, B, C or D) were calculated for each subject (mathematics, foreign language and primary language) and plotted using the effects R library [37].

The association between PGS and behavioral and emotional problems was tested using linear-mixed effects models. The CBCL and Conners’ parent rating scales were used as continuous variables and square-root transformed because of skewness.

All analyses were adjusted for age, sex, socioeconomic status (SES) and 20 genetic PC as fixed effects, as well as school as random effects to account for the multilevel nature of the data, considering each genotyping wave separately. SES was calculated using the Hollingshead Two-Factor Index based on parent’s education and occupation [38]. Results from each wave were then pooled using fixed-effects inverse variance weighted meta-analyses with the *meta* R library [39]. PGS P-values were corrected for multiple comparisons using Benjamini–Hochberg False Discovery Rate (FDR) method (adj-*P* < 0.05). Pairwise correlations between PGS from concordant and discordant genomic partitions were calculated using the non-parametric spearman rank correlation coefficient.

#### Mediation analyses

The effect of ADHD or ASD symptoms as mediators of the relationship between PGS of interest and school performance was assessed using a mediation analyses. We first estimated the effect of PGS on the mediator by using linear models and the effect of the mediator on school performance using ordinal mixed-effect models. If significant associations were detected in these two models (*P* < 0.05), the direct effect of PGS on school performance after taking into account the effect of the mediator was obtained using ordinal mixed-effect models. Mediators were square-root transformed to approximate normal distribution in all analyses where they were outcomes. All analyses were adjusted for age, sex, SES and 20 genetic PC as fixed effects, as well as school as random effects, considering each genotyping wave separately. Results from each wave were then pooled using fixed-effects inverse variance weighted meta-analyses with the *meta* R library [37]. After Bonferroni multiple testing correction (taking into account three subjects and two genomic partitions included in the mediation analysis for each disorder, *P*-value < 8.3e−03), we considered full mediation when the direct effect of the PGS on school performance becomes non-significant after taking into account the mediator and partial mediation when the direct effect remains significant, but to an attenuated degree.

#### Partitioned heritability

SNP-based heritability (h^2^_SNP_) estimates for ADHD and ASD were calculated using LDSC v1.0. [40, 41] for the three genome partitions described above: variants not associated with EA, variants with concordant and variants with discordant effects in EA. SNP-based heritability was computed for each annotation file using the GWAS summary statistics of ADHD and ASD [3, 13] and data from 1000G as reference panel [33]. Analyses were restricted to Hapmap3 SNPs and the major histocompatibility complex region was excluded. To improve the model performance, independent LD scores from the full baseline model v2.2 were used, which consist in a full annotation column and 97 independent functional annotations, which are available at LDSC repository (https://zenodo.org/record/7768714#.ZGaHZOzP3R1). Enrichment of genome-wide significant hits (*P* < 5e−08) of ADHD or ASD in the genome partitions described above were calculated using a Chi-square test and Manhattan plots were obtained using the *qqmap* R package.

#### Cell-type specific partitioned heritability

Cell-type specific analysis of partitioned heritability was run on LDSC v1.0. [40, 41] to explore the biological underpinnings of ADHD and ASD genomic partitions with significant h^2^_SNP_. These analyses were performed on new annotation files created from the intersection of each genomic partition and 13 brain-related tissues (Brain GTEx) [42] and three brain cell-type annotation files (neuron, astrocytes and oligodendrocytes) [43]. Reference annotation files were constructed as previously described [44] using two approaches: (i) “control” files - the intersection between each genomic partition and the provided control files in LDSC which include all genes in each study, and (ii) “anti-target” files – the intersection between the cell-type or brain specific annotation and the variants not included in the corresponding genomic partition. This “anti-target” analysis compares specific cell-type enrichment within a particular genomic partition and outside of this genomic partition, enabling us to assess the influence of EA on a particular cell-type. For each annotation file, we obtained an enrichment score (proportion of total SNPs in an annotation/percentage of the h2SNP) and then evaluated whether this cell-type enrichment was higher than the one associated to the “control” or “anti-target” enrichment. This was done by applying the LD score regression to specifically expressed genes (LDSC-SEG) approach using the --h2-cts argument [42]. *P*-values were corrected for multiple comparisons using FDR method (adj-*P* < 0.05).

#### Genetic covariance

Annotation-stratified genetic covariance between ADHD or ASD with multiple disorders and traits was assessed with GNOVA (https://github.com/xtonyjiang/GNOVA) [45], which calculates the genetic covariance between two phenotypes considering the LD structure and sample overlap. Covariance analyses considering the overall set of SNPs for ADHD or ASD were also performed [3, 13]. Available summary statistics from GWAS on 14 neuropsychiatric disorders, cognition and personality traits previously correlated with ADHD and/or ASD were considered, including ADHD [3], ASD [13], anorexia nervosa [46], antisocial behavior [47], anxiety disorder, bipolar disorder [48], extraversion [49], intelligence quotient (IQ) [50], loneliness and isolation, major depression [51], risk tolerance [52], schizophrenia [53], subjective well-being [54] and substance use disorder (SUD) [55]. GWAS summary statistics for anxiety disorder as well as loneliness and isolation were downloaded from Ben Neale UK Biobank GWAS analysis webpage (http://www.nealelab.is/uk-biobank). P-values were corrected for multiple comparisons using FDR (adj-*P* < 0.05).

## Results

### Contribution of the genetic liability for ADHD, ASD and EA on school performance

PGS for educational attainment (PGS_EA_) were associated with higher odds of better performance in mathematics (OR = 1.43, 95% CI = 1.35–1.52; adj-*P* = 1.0e−28), foreign language (OR = 1.32, 95% CI = 1.24–1.41; adj-*P* = 1.0e−17) and primary language (OR = 1.40, 95% CI = 1.32–1.50; adj-*P* = 2.1e−24), while PGS for ADHD showed the opposite pattern of associations, with PGS_ADHD_ associated with worse performance in all three subjects: mathematics (OR = 0.81, 95% CI = 0.76–0.85; adj-*P* = 2.3e−12), foreign language (OR = 0.81, 95% CI = 0.76–0.85; adj-*P* = 2.8e−12) and primary language (OR = 0.80, 95% CI = 0.76–0.85; adj-*P* = 4.5e−12) (Table 1 and Supplementary Table 1). No association was found between PGS for ASD (PGS_ASD_) and school performance.

**Table 1:** Associations between genome-wide polygenic scores (PGS) for ADHD and ASD dissected by its relationship with EA and school performance in mathematics, foreign language and primary language.

	Subject	PGS^a^	OR (95% CI)	Statistic	*P*-value	adj *P*-value^b^	R^2c^
EA	Mathematics	EA	1.43(1.35–1.52)	11.42	**3.2E−30**	**1.0E−28**	4.28%
	Foreign language	EA	1.32(1.24–1.41)	8.84	**9.5E−19**	**1.0E−17**	2.68%
	Primary Language	EA	1.40(1.32–1.50)	10.46	**1.3E−25**	**2.1E−24**	3.30%
ADHD	Mathematics	ADHD	0.81(0.76–0.85)	−7.23	**5.0E−13**	**2.3E−12**	2.01%
		ADHD_noEA	0.87(0.82–0.92)	−4.78	**1.7E−06**	**3.0E−06**	0.68%
		ADHD_EA	0.80(0.75–0.85)	−7.46	**9.0E−14**	**4.9E−13**	2.20%
		ADHDdiscordant	0.77(0.72–0.81)	−8.79	**1.5E−18**	**1.3E−17**	2.63%
		ADHDconcordant	1.03(0.97–1.09)	0.94	0.35	0.41	0.12%
	Foreign language	ADHD	0.81(0.76–0.85)	−7.19	**6.7E−13**	**2.8E−12**	1.86%
		ADHD_noEA	0.85(0.80–0.90)	−5.50	**3.7E−08**	**7.7E−08**	0.93%
		ADHD_EA	0.84(0.79–0.89)	−5.98	**2.2E−09**	**5.3E−09**	1.39%
		ADHDdiscordant	0.81(0.76–0.86)	−6.84	**7.7E−12**	**2.5E−11**	1.80%
		ADHDconcordant	1.00(0.95–1.06)	0.10	0.92	0.95	0.04%
	Primary Language	ADHD	0.80(0.76–0.85)	−7.10	**1.2E−12**	**4.5E−12**	1.84%
		ADHD_noEA	0.85(0.80–0.90)	−5.49	**4.0E−08**	**7.8E−08**	1.02%
		ADHD_EA	0.82(0.77–0.87)	−6.60	**4.2E−11**	**1.3E−10**	1.72%
		ADHDdiscordant	0.77(0.72–0.82)	−8.33	**7.8E−17**	**5.2E−16**	2.40%
		ADHDconcordant	1.07(1.01–1.13)	2.19	**0.028**	**0.042**	0.24%
ASD	Mathematics	ASD	0.96(0.91–1.02)	−1.39	0.17	0.21	0.20%
		ASD_noEA	0.95(0.90–1.01)	−1.64	0.10	0.13	0.25%
		ASD_EA	1.00(0.94–1.06)	0.01	0.99	0.99	0.20%
		ASDdiscordant	0.83(0.78–0.88)	−6.30	**3.0E−10**	**7.5E−10**	1.95%
		ASDconcordant	1.17(1.11–1.24)	5.38	**7.4E−08**	**1.3E−07**	1.23%
	Foreign language	ASD	0.94(0.89–1.00)	−2.08	0.04	0.05	0.19%
		ASD_noEA	0.94(0.89–1.00)	−2.03	0.042	0.058	0.14%
		ASD_EA	0.97(0.91–1.02)	−1.13	0.26	0.32	0.19%
		ASDdiscordant	0.84(0.79–0.89)	−5.66	**1.5E−08**	**3.3E−08**	1.12%
		ASDconcordant	1.11(1.04–1.17)	3.39	**6.9E−04**	**1.1E−03**	1.18%
	Primary Language	ASD	0.98(0.93–1.04)	−0.55	0.58	0.64	0.04%
		ASD_noEA	0.99(0.93–1.05)	−0.40	0.69	0.73	0.04%
		ASD_EA	0.98(0.92–1.03)	−0.82	0.41	0.47	0.02%
		ASDdiscordant	0.82(0.77–0.87)	−6.55	**5.9E−11**	**1.6E−10**	1.64%
		ASDconcordant	1.15(1.09–1.23)	4.66	**3.1E−06**	**5.2E−06**	1.28%

Number of variants included in each PGS: 10,101,242 in EA; 6,774,224 in ADHD; 5,462,609 in ADHD_noEA; 1,208,840 in ADHD_EA; 424,130 in ADHDdiscordant; 781,844 in ADHDconcordant; 9,112,386 in ASD; 5,905,297 in ASD_noEA; 1,258,653 in ASD_EA; 683,565 in ASDdiscordant; 572,799 in ASD concordant.

^a^Analysis of PGS of ADHD and ASD using the whole set of genetic variants (EA, ADHD or ASD) and the four subsets of genetic variants: SNPs no associated with EA (“_noEA” suffix), SNPs associated with EA (“_EA” suffix), SNPs associated with EA showing an opposite direction of effects (“discordant”) and SNPs showing concordant direction of effects (“concordant”).

^b^FDR-corrected significant associations (adj *P*-value < 0.05) are marked in bold.

^c^Mean of R^2^ calculated in each genotyping wave as the increase in Nagelkerke’s pseudo-R^2^ between an ordinal model with and without the PGS variable.

**Fig. 2:**
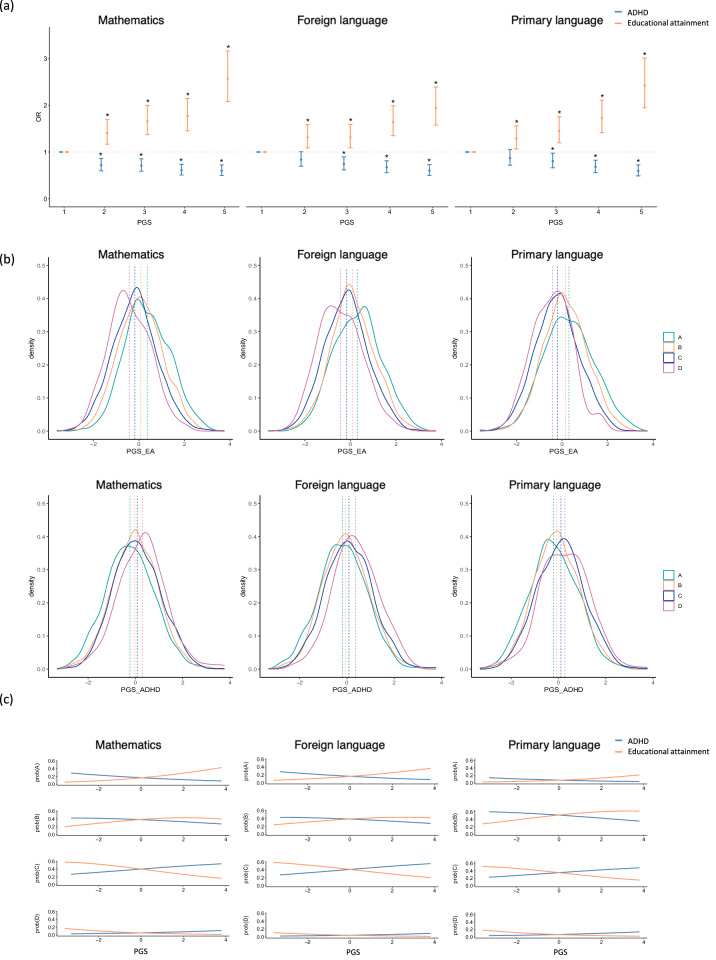
Contribution of genome-wide polygenic scores (PGS) for educational attainment (PGS_EA_) and ADHD (PGS_ADHD_) to school performance in three different subjects: mathematics, foreign language and primary language. **a** Quantile plots of meta-analysis odds ratios (OR) with 95% confidence intervals for PGS_EA_ (in orange) and PGS_ADHD_ (in blue). The target sample was divided into quintiles, and school performance of each quintile was compared to the first quintile using ordinal mixed-effect models with age, sex, SES and 20 principal genetic components as fixed effects and school as random effects. Significant comparisons (*P*_FDR_ < 0.05) are indicated with an asterisk; **b** Density plots of the contribution of PGS_EA_ and PGS_ADHD_ to school performance grades for the three subjects under study. **c** Probability plots showing the probability of reaching each school performance grade (A, B, C or D) in each of the subjects. Per each subject we show four sections of probability corresponding to each school performance grade according to the PGS_EA_ (in orange) and PGS_ADHD_ (in blue).

PGS comparisons across ranked quintiles showed that the odds for better performance in children at the highest quintile for PGS_EA_ was, on average, over twice than in children in the first quintile for the three subjects (Fig. 2a and Supplementary Table 2a). Conversely, children at higher quintiles for PGS_ADHD_ showed lower odds for better performance than children in the first quintile (Fig. 2a and Supplementary Table 2b). Consistently, the probability of reaching better scores (A or B) increased with higher PGS_EA_ or lower PGS_ADHD_. Likewise, the probability of reaching worse scores (C or D) increased with higher PGS_ADHD_ or lower PGS_EA_ (Fig. 2b, c).

### Polygenic dissection of the contribution of ADHD and ASD to school performance by their relationship with EA

We constructed PGS for these neurodevelopmental disorders with four subsets of SNPs based on to their contribution to EA, including variants not associated with EA (PGS_ADHD_noEA_/PGS_ASD_noEA_) and variants associated with EA (PGS_ADHD_EA_/PGS_ASD_EA_), which were divided in concordant (PGS_ADHDconcordant_/PGS_ASDconcordant_), showing consistent direction of effect in EA and ADHD/ASD, and discordant variants (PGS_ADHDconcordant_/PGS_ASDconcordant_), showing opposite direction of effect in EA and ADHD/ASD (Fig. 1).

**Fig. 3:**
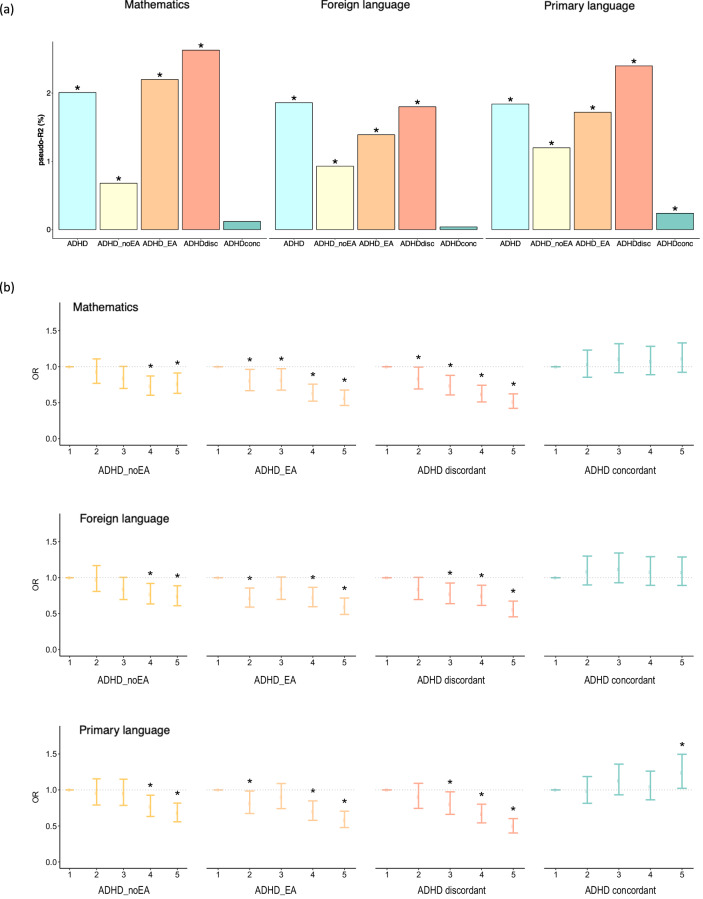
Contribution of genome-wide polygenic scores for ADHD (PGS_ADHD_) dissected by their relationship with EA to school performance in three different subjects: mathematics, foreign language and primary language. PGS_ADHD_ were constructed considering four subsets of SNPs: variants not associated with EA (*P*_EA_ > 0.05; PGS_ADHD_noEA_), variants associated with EA (*P*_EA_ < = 0.05; PGS_ADHD_EA_), variants showing concordant (PGS_ADHDconcordant_) and discordant (PGS_ADHDdiscordant_) direction of the effect in EA and ADHD. **a** Percentage of variance, as mean of Nagelkerke’s pseudo-R^2^ calculated in each genotyping wave, explained by PGS_ADHD_ according to the four partitions described above; **b** Quantile plots of meta-analysis odds ratios (OR) with 95% confidence intervals for PGS_ADHD_ according to the four partitions described above. The target sample was divided into quintiles and school performance of each quintile was compared to the first quintile using ordinal mixed-effect models with age, sex, SES and 20 principal genetic components as fixed effects and school as random effects. Significant comparisons (*P*_FDR_ < 0.05) are indicated with an asterisk.

Both PGS_ADHD_noEA_ and PGS_ADHD_EA_ were associated with worse school performance in the three subjects (adj-*P* < = 3.0e−06) (Table 1 and Supplementary Table 3). When focused on variants associated with EA, we found that PGS_ADHDdiscordant_ were associated with poor school performance in mathematics (OR = 0.77, 95% CI = 0.72–0.81; adj-*P* = 1.3e−17), foreign language (OR = 0.81, 95% CI = 0.76–0.86; adj-*P* = 2.5e−11) and primary language (OR = 0.77, 95% CI = 0.72–0.82; adj-*P* = 5.2e−16) (Table 1 and Supplementary Table 3). Conversely, PGS_ADHDconcordant_ showed the opposite direction of effect and were associated, to a lesser extent than PGS_ADHDdiscordant_, with better performance in primary language (OR = 1.07, 95% CI = 1.01–1.13; adj-*P* = 0.042) (Table 1 and Supplementary Table 3). PGS_ADHDdiscordant_ accounted for the largest proportion of the variance explained for school performance compared to the other PGS_ADHD_, ranging from 1.8% in foreign language to 2.6% in mathematics (Fig. 3a and Table 1). It explained on average over 28 times more variance for school performance than PGS_ADHDconcordant_ across the three subjects (Fig. 3a and Table 1).

PGS comparisons across ranked quintiles showed the expected trend of lower odds for better school performance in individuals with higher PGS_ADHD_ across all partitions but PGS_ADHDconcordant_ (Fig. 3b and Supplementary Table 3). The strongest effect was identified for PGS_ADHDdiscordant_, with children at the highest quintile doubling the odds for underperformance at school of children in the first quintile (Fig. 3b and Supplementary Table 3). Consistently, higher PGS_ADHDdiscordant_ decreased the probability of reaching an A or B score while the probability of reaching a C or D score increased in all three subjects (Supplementary Fig. 1). Besides, PGS_ADHDconcordant_ showed a trend towards the opposite direction of the association, with higher PGS_ADHDconcordant_ associated with higher probability of school success, especially in primary language, where children at the highest quintile showed 1.24 times better performance than children in the first quintile (Fig. 3b and Supplementary Table 3).

Then we assessed whether ADHD symptoms explained the associations between polygenic risk for ADHD and school performance. Both PGS_ADHDnoEA_ and PGS_ADHDdiscordant_ were associated with increased ADHD symptoms (*P* = 2.9e−12 and *P* = 7.1e−13, respectively) (Supplementary Table 4), and ADHD symptoms lower odds for better school performance in all subjects (*P* < 1.5e−121) (Supplementary Table 5). The effect of both PGS on school performance decreased after considering the effect of ADHD symptoms, suggesting that ADHD symptoms partially mediate the effect of PGS_ADHDdiscordant_ (from 16% in primary language to 37% in foreign language) and fully mediate the effect of PGS_ADHDnoEA_ (from 52% in primary language to 55% in mathematics) on school performance (Supplementary Tables 6 and 7).

**Fig. 4:**
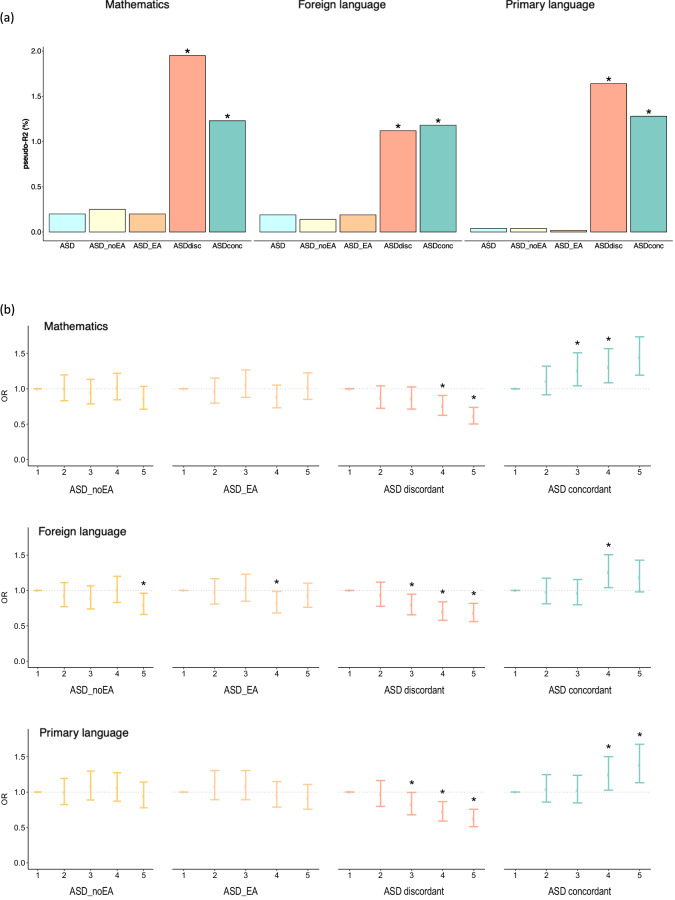
Contribution of genome-wide polygenic scores for ASD (PGS_ASD_) dissected by their relationship with EA to school performance in three different subjects: mathematics, foreign language and primary language. PGS_ASD_ were constructed considering four subsets of SNPs: variants not associated with EA (*P*_EA_ > 0.05; PGS_ASD_noEA_), variants associated with EA (*P*_EA_ < = 0.05; PGS_ASD_EA_), variants showing concordant (PGS_ASDconcordant_) and discordant (PGS_ASDdiscordant_) direction of the effect in EA and ASD. **a** Percentage of variance, as mean of Nagelkerke’s pseudo-R^2^ calculated in each genotyping wave, explained by PGS_ASD_ according to the four partitions described above; **b** Quantile plots of meta-analysis odds ratios (OR) with 95% confidence intervals for PGS_ASD_ according to the four partitions described above. The target sample was divided into quintiles and school performance of each quintile was compared to the first quintile using ordinal mixed-effect models with age, sex, SES and 20 principal genetic components as fixed effects and school as random effects. Significant comparisons (*P*_FDR_ < 0.05) are indicated with an asterisk.

When we subset the genetic liability for ASD based on its role in EA, PGS_ASD_ showed a different pattern of association with school performance than PGS_ADHD_. In line with the lack of association between PGS_ASD_ and school performance, no association was found either for PGS_ASD_noEA_ or PGS_ASD_EA_ (Table 1 and Supplementary Table 3). However, the genetic liability with concordant and discordant effects between ASD and EA showed significant association with school performance with opposite direction of effect. PGS_ASDdiscordant_ were associated with poor school performance in mathematics (OR = 0.83, 95% CI = 0.78–0.88; adj-*P* = 7.5e−10), foreign language (OR = 0.84, 95% CI = 0.79–0.89; adj-*P* = 3.3e−08) and primary language (OR = 0.82, 95% CI = 0.77–0.87; adj-*P* = 1.6e−10) (Fig. 4a, Table 1 and Supplementary Table 3). Whereas, PGS_ASDconcordant_ were associated with better performance in mathematics (OR = 1.17, 95% CI = 1.11–1.24; adj-*P* = 1.3e−07), foreign language (OR = 1.11, 95% CI = 1.04–1.17; adj-*P* = 1.1e−03) and primary language (OR = 1.15, 95% CI = 1.09–1.23; adj-*P* = 5.2e−06) (Table 1 and Supplementary Table 3). PGS_ASDdiscordant_ accounted for the largest proportion of the variance explained for school performance (from 1.12% in foreign language to 1.95% in mathematics) followed by PGS_ASDconcordant_ (from 1.18% in foreign language to 1.28% in primary language) (Fig. 4a and Table 1).

PGS comparisons across ranked quintiles showed that children at the highest quintile for PGS_ASDdiscordant_ had lower odds for better performance than children in the first quintile (*P* < = 5.3e−05), while children at higher quintiles for PGS_ASDconcordant_ showed better performance than children in the first quintile in all three subjects (*P* < = 0.018) (Fig. 4b and Supplementary Table 2c). Consistently, the probability of reaching an A or B score increased with higher PGS_ASDconcordant_ or lower PGS_ASDdiscordant_, while the probability of reaching a C or D score increased with higher PGS_ASDdiscordant_ or lower PGS_ASDconcordant_ (Supplementary Fig. 2).

Also, ASD symptoms emerged as partial mediators of the effect of the ASD genetic liability on school performance. PGS_ASDconcordant_ and PGS_ASDdiscordant_ were significantly associated with increased ASD symptoms (*P* = 6.3e−04 and *P* = 1.1e−4, respectively) (Supplementary Table 4), and ASD symptoms with lower school performance in all three subjects (*P* < = 4.2e−33) (Supplementary Table 5). The effect of PGS_ASDdiscordant_ on poor school performance decreased when taking into account the effect of ASD symptoms, suggesting that they mediate from 6% to 9% of the effect of PGS_ASDdiscordant_ on school performance (Supplementary Tables 6 and 7). By contrast, for PGS_ASDconcordant_ we found evidence of inconsistent mediation or suppressor effects [56, 57] that occur when two predictors (PGS_ASDconcordant_ and ASD symptoms) with different direction of effect on the outcome (school performance) are simultaneously considered. PGS_ASDconcordant_ and ASD symptoms operate consistently as mutual suppressor for school performance, meaning that taking into account the effect of ASD symptoms into the regression equation increases the positive effect of PGS_ASDconcordant_ on school performance (from 13% in mathematics to 24% in foreign language) (Supplementary Tables 6 and 7).

When stratifying by sex, we found no differences on the effect of the genetic liability for ADHD or ASD dissected by its relationship with EA on school performance (*P*_interaction_ > 0.05) (Supplementary Table 8).

Given the significant negative correlation found between PGS_ADHDdiscordant_ and PGS_ASDconcordant_ (ρ = −0.29, *P* = 8.8e−82; Supplementary Fig. 3), a sensitivity analysis was performed to further assess the effect of the overlapping variation between ADHD and ASD on school performance. PGS constructed on the subset of shared variants (PGS_ADHDshared_ and PGS_ASDshared_) were associated with school performance with opposite direction of the effect in the three subjects under study. While PGS_ADHDshared_ contributed to poor school performance, PGS_ASDshared_ were associated with better performance (Supplementary Table 9), results supporting that the same set of SNPs may drive the discordant association of both disorders with school performance.

### Polygenic dissection of the contribution of ADHD and ASD to childhood psychopathology by their relationship with EA

**Fig. 5:**
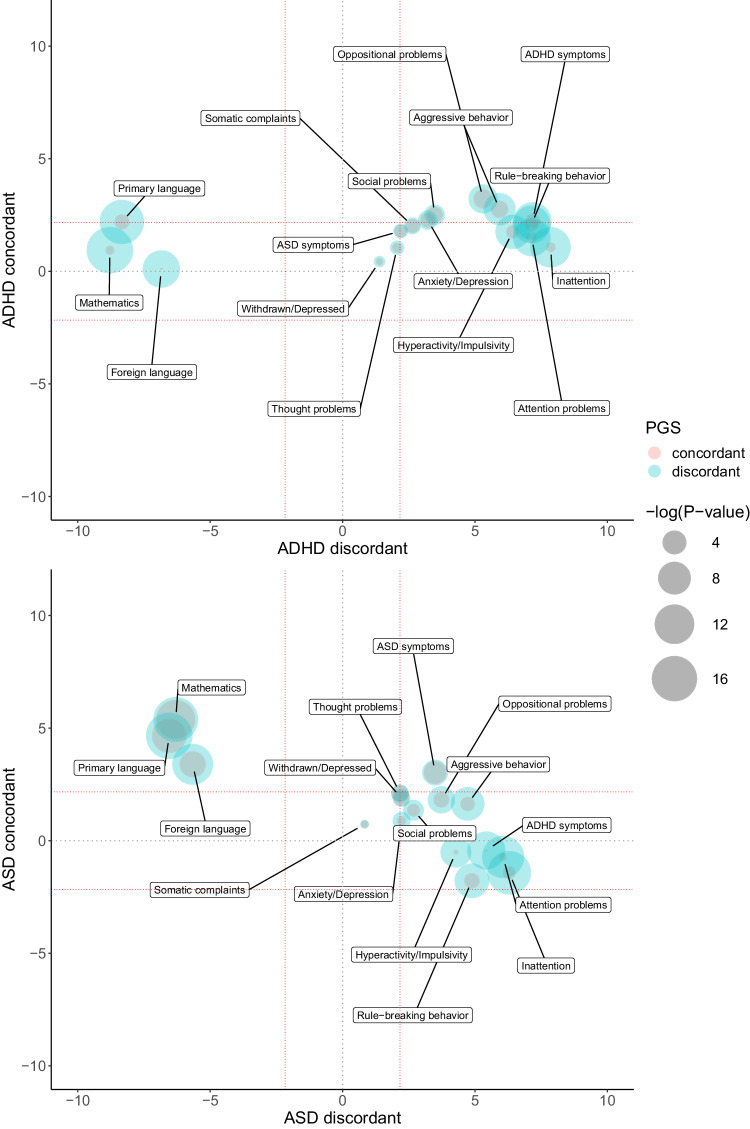
Dissection of childhood psychopathology by genome-wide polygenic scores (PGS) constructed on variants showing concordant or discordant direction of the effect in EA and ADHD or ASD. Z-scores from regression analyses for school performance in the three subjects, CBCL scales or Conners’ parent rating scales and (**a**) PGS_ADHDdiscordant_ (x axis) and PGS_ADHDconcordant_ (y axis) or (**b**) PGS_ASDdiscordant_ (x axis) and PGS_ASDconcordant_ (y axis). Point size corresponds to -log10(*p*-value) with concordant PGS in red and discordant PGS in blue. Red dotted lines indicate significant results after 5% FDR correction.

The majority of behavioral and emotional problems showed a distinct pattern of association with the genetic liability for ADHD and ASD by their role in EA, being most of them positively associated with the subset of variants with discordant, but not concordant, direction of effect (i.e., inattention, hyperactivity/impulsivity or rule-breaking behavior) (Fig. 5). Many of those displaying association with discordant variation were also associated with PGS_noEA_. Also, some traits were associated with the genetic liability for ADHD (i.e., ADHD symptoms, oppositional problems, social problems and aggressive behavior) or ASD (i.e., ASD symptoms) regardless of EA, with concordant effect sizes being generally smaller than those for the discordant set of variants or variants not associated with EA (Fig. 5 and Supplementary Table 10).

### Partitioned heritability

Partitioned heritability estimates for ADHD showed significant h^2^_SNP_ enrichment in ADHD_discordant_ and ADHD_noEA_ variants, but negative h^2^_SNP_ in the ADHD_concordant_ SNP set, indicating that most of the SNPs in this group have null effects on ADHD or suggesting a model misspecification (Supplementary Table 11). Consistently, we found enrichment of genome-wide significant hits for ADHD [3] within the ADHD_discordant_ and ADHD_noEA,_ (*P* < 2.2e−16), but not the ADHD_concordant_, SNP subsets (Supplementary Fig. 4a–c). For ASD, only the ASD_concordant_ subset of variants showed significant h^2^_SNP_ enrichment (Supplementary Table 11) and none of the SNP partitions showed enrichment of ASD hits [13] (Supplementary Fig. 4d–f).

To further explore the biological underpinnings of ADHD and ASD genomic partitions with significant h^2^_SNP_ we used partitioned heritability and LD score regression applied to specifically expressed genes (LDSC-SEG) [42]. When we compared specific cell-type enrichment inside and outside a particular genomic partition considering the “anti-target” reference annotations, we found that the ADHD_discordant_ partition, but not the ADHD_noEA_, was enriched by ADHD h^2^_SNP_ in all studied cell-types and brain areas (Supplementary Fig. 5 and Table 12). We also found enrichment in ASD h^2^_SNP_ within the ASD_concordant_ partition from neurons and four brain areas (i.e., anterior cingulate cortex, cerebellum, frontal cortex, hypothalamus; Supplementary Fig. 5 and Table 12). No significant results, were observed when comparing the enrichment of h^2^_SNP_ with the “control” annotation for any partition (Supplementary Table 12).

### Genetic covariance between ADHD and ASD and related disorders and traits dissected by their relationship with EA

ASD displayed positive covariance with ADHD_noEA_ and ADHD_concordant_, while ADHD had positive covariance with ASD_discordant_ and negative covariance with ASD_concordant_, confirming the opposite genetic overlap between both disorders and EA (Supplementary Table 13 and Fig. 6).

When we assessed the genetic covariance between ADHD or ASD and a set of neuropsychiatric disorders, cognition and personality traits, we found similar patterns for most of the traits and SNP partitions. Estimated covariances for variants not associated with EA were similar to genetic correlations previously described for ADHD or ASD across the whole genome [3, 13], except for IQ. The majority of tested phenotypes showed opposite patterns of genetic covariance within the sets of variants with concordant or discordant effects for both disorders. Anorexia nervosa, IQ and subjective well-being displayed positive covariance with concordant but negative covariance with discordant genetic load. Conversely, SUD, loneliness and isolation and antisocial behavior displayed positive covariance with discordant and negative covariance with concordant genetic variation for both disorders. In contrast, for several traits the genetic covariance with ADHD (i.e., risk tolerance or schizophrenia) or ASD (i.e., schizophrenia) did not depend on EA, and others had a significant covariance only with either the concordant (i.e., bipolar disorder) or the discordant (i.e., anxiety or major depression) set of variants according to their effect on EA (Supplementary Table 13 and Fig. 6).

## Discussion

Our results reveal that, despite sharing a common genetic architecture, the genetic liability for ADHD and ASD show differences in their association with school performance and early manifestation of psychopathology by their relationship with EA. This highlights the potential of leveraging overlapping polygenic signals with EA to dissect clinical and genetic heterogeneity in mental health, as described for schizophrenia [58].

The strong association between PGS for adult EA and better school grades in childhood and adolescence confirms the lifetime nature of the EA genetic background, supporting that school performance is an early life intermediate phenotype for future outcomes in adulthood. These results align with previous evidence of higher polygenic scores for EA associated with speech and reading skills even before school, as well as with loftier academic aspiration and attainment extending into adulthood [8].

We confirm the strong and inverse association between polygenic ADHD risk and school performance [59], which involves genetic variation shared with EA, especially when variants with discordant effects in both traits were considered. We found that the genetic liability for ADHD not associated with EA also impacts on school performance, suggesting that observed genetic associations between ADHD and poor academic outcomes may not only reflect shared genetic variation with EA, but also specific ADHD genetic liability, independently of EA. These results are in line with findings where ADHD associations with literacy-related impairments involve genetic variation shared with, but also independent of, EA [59]. Interestingly, the ADHD_noEA_ genomic partition was found associated with higher IQ, as well as with several psychiatric disorders positively correlated with EA and/or IQ in previous studies, such as ASD, anorexia nervosa, schizophrenia or bipolar disorder [7, 50, 60, 61]. These apparent contradictory findings could be explained by the fact that school performance is not only explained by cognitive traits but also by other factors such as self-perceived abilities, psychopathology or well-being, even after accounting for IQ [6, 62, 63]. In addition, apart from the genetic liability associated with high IQ, ADHD_noEA_ may also account for noncognitive factors, such as ADHD symptoms, which can change across the lifespan [64], and might affect early-life school outcomes without having an effect on later-life educational attainment. We found that this genomic partition also correlates negatively with subjective well-being and positively with SUD, loneliness and isolation, antisocial behavior, risk tolerance, anxiety or depression, that may contribute negatively on school performance independently of IQ. Interestingly, similar results were described for late-life EA, where beyond heritable cognitive skills, there are noncognitive influences that have a polygenic architecture, impact on EA and correlate with personality traits and increased risk for certain psychiatric disorders [7].

ADHD shows negative genetic correlation with EA, with the vast majority of risk variants being associated with low levels of EA [3]. Consistently, we found that the overlapping genetic variation with discordant effects in both phenotypes has the strongest negative effect on school performance, which is partially mediated by ADHD symptoms. It also accounts for most of the ADHD genetic background described to date [3], and is associated with higher rates of children psychopathology. Conversely, concordant variation between ADHD and EA has no impact on school performance and contributes, to less extent than discordant variation, to ADHD symptoms and externalizing behaviors. These results confirm that a large proportion of ADHD risk loci is shared with EA and has opposite direction of the effect, and provide further evidence on the adverse effects of ADHD on long-term academic outcomes [65].

The polygenic association of ASD and school performance was to a large extent attributable to genetic effects that are shared with EA. Both the concordant and discordant variation that make up the genetic load for EA have polygenic contributions to school performance, which are partially explained by ASD symptoms but shows divergent patterns of genetic effects. While genetic variation with discordant effects in ASD and EA was associated with impaired performance at school and early manifestation of psychopathology, PGS for ASD constructed on variants with concordant effects were associated with improved school performance and ASD symptoms, but not with other emotional or behavioral problems. This complex relationship may underlie the variability in academic achievement observed across the autism spectrum [23], strongly supports that ASD is genetically heterogeneous and demonstrates that ASD and EA share many genetic loci but without a clear pattern of sign concordance. Consistently, recent findings showed that most genetic variants associated with ASD are shared with EA, with both positive and negative direction of the effect [11], which may explain the lack of association found between school performance and genome-wide PGS for ASD. The combination of ASD discordant and concordant variation with opposite effects in school performance may result in the cancellation of the association signal, which aligns with observational studies showing that academic achievement in ASD is more variable than in ADHD [23], and strongly supports our study design that leverages EA genetic data to detect opposite polygenic effects that would otherwise be missed.

We also found that a great proportion of the ASD genetic liability is not associated either with EA nor with school performance. Genetic variants included in this ASD_noEA_ genomic partition may contribute to ASD symptoms different from cognition with no impact on school performance, such as difficulties in social interaction. This idea was supported by the analysis of genomic covariance, where we found that this genomic partition was negatively correlated with extraversion but not associated with IQ. For most of the psychiatric disorders and related traits, the pattern of genetic covariance was opposite for concordant and discordant overlapping variation, the latter being more similar to that previously described for ADHD or ASD [2, 3, 13]. These results align with recent findings reporting association between poor school achievement and greater risk of a subsequent mental disorder, except for eating disorders, where the association was of similar magnitude but in the opposite direction [66]. Our findings are consistent with the epidemiological and genetic correlations described between ADHD or ASD and other psychiatric conditions [2] and provide further evidence that genetic data on EA, in aggregate, aid to identifying individuals at higher risk not only for poor school performance but also for worse disease course and clinical outcomes. The genetic covariance results also suggest that concordant genomic partitions are associated with positive outcomes, as reflected by positive genetic covariance with subjective well-being and negative genetic covariance with different psychiatric disorders and traits (i.e., SUD, antisocial behavior or loneliness and isolation). However, this pattern of association was not observed in schizophrenia, bipolar disorder and ASD, which are positively correlated with concordant partitions. This is in line with a recent study supporting that bipolar disorder, schizophrenia and ASD may belong to the same psychopathology factor (i.e., thought problems factor) at the genomic level [67].

The main strength of our study is to disentangle the polygenic effects of ADHD and ASD on school performance using a third trait, EA, allowing us to better understand their polygenic contribution and early manifestation of psychopathology in a deeply phenotyped population-based cohort. However, several considerations need to be contemplated. First, we found that discordant polygenic signatures for both disorders correlate with early manifestation of psychopathology and poor school performance, which confirms genetic influences transcending diagnostic boundaries and suggests that prodromal symptoms may negatively affect school performance. However, we did not infer causality and cannot exclude reverse causation, where poor school achievement increases the risk for psychopathology later in life [68–72]; Second, although our results are robust when accounting for potential confounders, including school and SES, we cannot discard residual confounding by other factors, such as treatment [73], meaning that we may underestimate the negative polygenic effect of ADHD and ASD on school performance; Third, the main findings held across the three subjects under study, which aligns with previous results on population-wide registers [73, 74]. Strikingly, there was also a trend towards better performance in primary language for ADHD concordant variation. Further analyses in larger samples will be needed to confirm whether this association is subject specific or can be extended to performance in foreign language or mathematics; Fourth, results from ADHD partitioned SNP heritability should be considered with caution since negative h^2^_SNP_ for the ADHD_concordant_ genomic partition could indicate a model misspecification; Finally, there are consistent evidences indicating that EA and school performance are influenced by demographic and indirect genetic effects [75]. Educational outcomes are particularly susceptible to bias arising from assortative mating or dynastic effects when studied in unrelated individuals, which may lead to inaccurate estimations of direct genetic effects [76–79]. Although the aim of this study was to dissect the polygenic contribution of ADHD and ASD to school performance by its relationship with EA, rather than estimating overall genetic effects, our results should be interpreted with caution in light of these population mechanisms. Further family-based study designs accounting for these population phenomena are required to provide more accurate genetic associations as well as to assess indirect genetic effects on school performance in the offspring.

In conclusion, we provide evidence showing that school performance is an early life intermediate phenotype associated with adult EA. By dissecting the genetic liability of ADHD and ASD by their effect on EA, we disentangled their polygenic effects on school performance and early manifestation of psychopathology, and provided new insights into overlapping genetic signatures with other comorbid disorders and traits. Overall, our findings expand on previous studies and strongly support the usage of the genetic load for EA to deepen insights into the genetic relationship between these neurodevelopmental disorders and school performance, to fill the gap of knowledge of the biological mechanisms underlying educational outcomes and to target more vulnerable individuals for early interventions.

## Supplementary information

Supplementary figures

Supplementary tables

## Data Availability

Raw data from this article is not publicly available because of limitations in ethical approvals and the summary data will be available from the corresponding autor upon reasonable request.
