# Supplementary material for: Dissecting the polygenic contribution of attention-deficit/hyperactivity disorder and autism spectrum disorder on school performance by their relationship with educational attainment
Source: Mol Psychiatry. 2024 May 23;29(11):3503–15. doi: 10.1038/s41380-024-02582-w (PMC11540845; doi:10.1038/s41380-024-02582-w)
Supplement: Supplementary file 1 — Supplementary figures [file 41380_2024_2582_MOESM1_ESM.docx]

**Dissecting the polygenic contribution of attention-deficit/hyperactivity disorder and autism spectrum disorder on school performance by their relationship with educational attainment**

**Supplementary Figures:**

Supplementary Figure 1. Contribution to school performance of genome-wide polygenic scores for ADHD considering variants associated with EA and showing concordant (PGS_ADHDconordant_) and discordant (PGS_ADHDdiscordant_) direction of the effect in ADHD and EA ……………………………………….… 2

Supplementary Figure 2. Contribution to school performance of genome-wide polygenic scores for ASD considering variants associated with EA and showing concordant (PGS_ASDconcordant_) and discordant (PGS_ASDdiscordant_) direction of the effect in EA and ASD…………………………………………….… 3

Supplementary Figure 3. Pairwise correlations between PGS from concordant and discordant genomic partitions ……………………………………………………………………………………………..…………………….. 4

Supplementary Figure 4. Manhattan plots for the ADHD and ASD subsets of variants based on their relationship with EA ……………………………………………………………………………………………..…………… 5

Supplementary Figure 5. Results from the partitioned heritability analysis with LDSC using “anti-target” reference annotations ……………….…………….…………….…………….…………….…………….………….. 9

Supplementary Figure 6. Annotation-stratified genetic covariance between ADHD or ASD and related neuropsychiatric disorders, cognition and personality traits………………………………………….11

**SUPPLEMENTARY FIGURES**

**Supplementary Figure 1. Contribution to school performance of genome-wide polygenic scores for ADHD considering variants associated with EA and showing concordant (PGS_ADHDconordant_) and discordant (PGS_ADHDdiscordant_) direction of the effect in ADHD and EA.** (a) Density plots of the contribution of the different PGS_ADHD_ to school performance grades; (b) Probability plots showing the probability of reaching each school performance grade (A, B, C or D) in each of the subjects. Per each subject we show four sections of probability corresponding to each school performance grade according to the PGS_ADHDconcordant_ (in yelow) or PGS_ADHDdiscordant_ (in green).

**Supplementary Figure 2. Contribution to school performance of genome-wide polygenic scores for ASD considering variants associated with EA and showing concordant (PGS_ASDconcordant_) and discordant (PGS_ASDdiscordant_) direction of the effect in EA and ASD.** (a) Density plots of the contribution of the different PGS_ASD_ to school performance grades; (b) Probability plots showing the probability of reaching each school performance grade (A, B, C or D) in each of the subjects. Per each subject we show four sections of probability corresponding to each school performance grade according to the PGS_ASDconcordant_ (in yellow) or PGS_ASDdiscordant_ (in green).

**Supplementary Figure 3. Pairwise correlations between PGS from concordant and discordant genomic partitions.** Inside each cercle the corresponding non-parametric spearman rank correlation coefficient. All correlations shown are nominally significant (P<0.05).


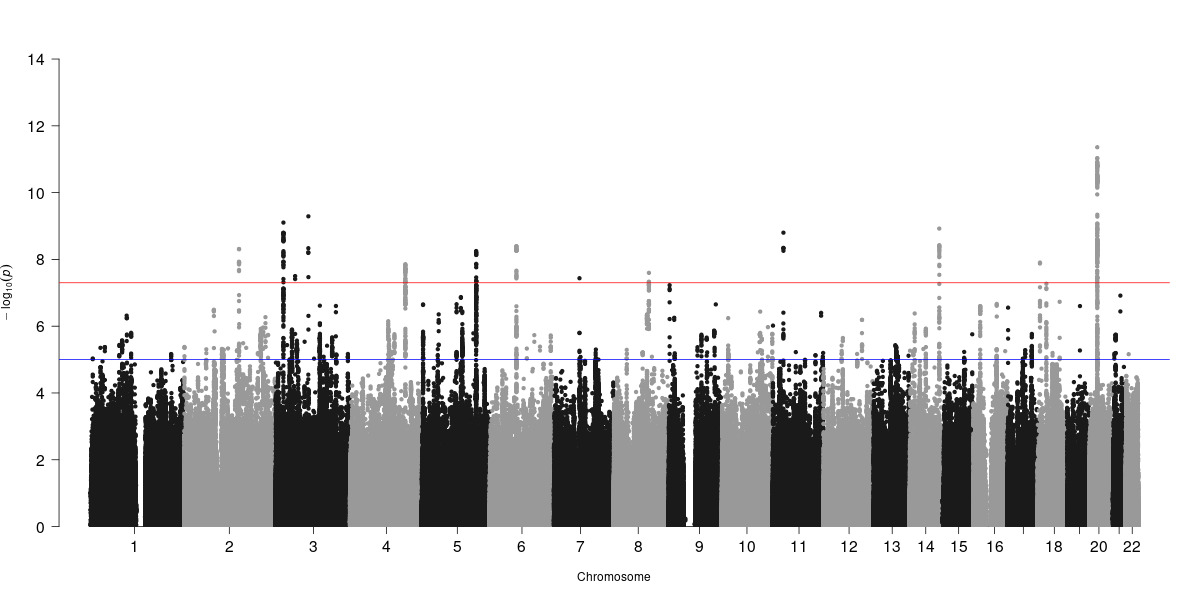


(a)

ADHDnoEA

-log(p-value)

Chromosome

(c)


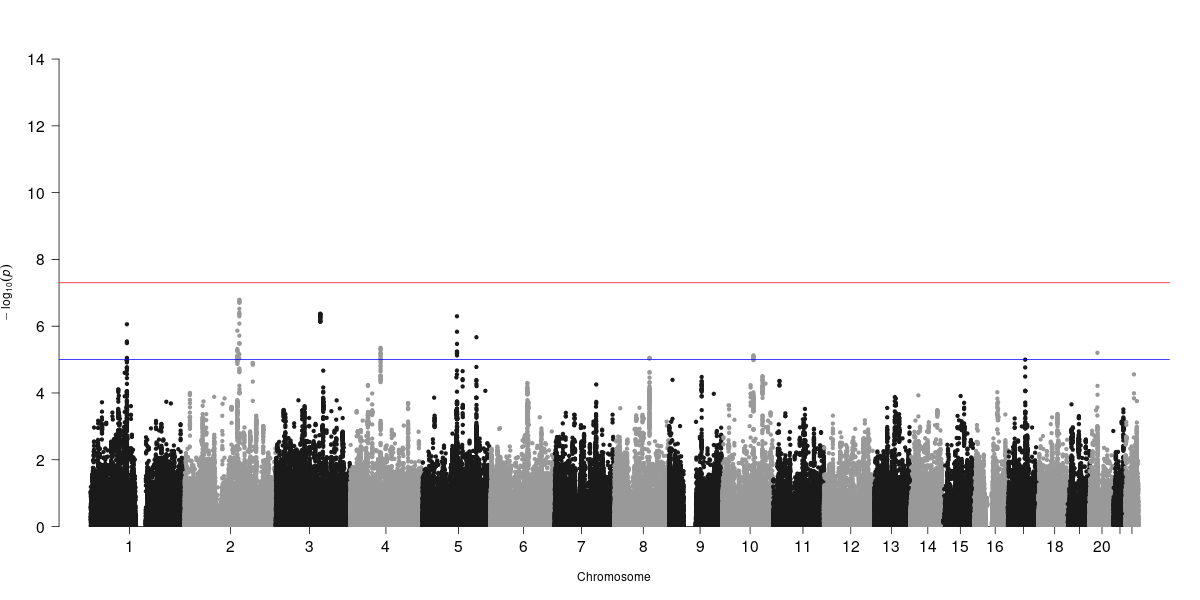


ADHDconcordant

-log(p-value)

Chromosome

(b)


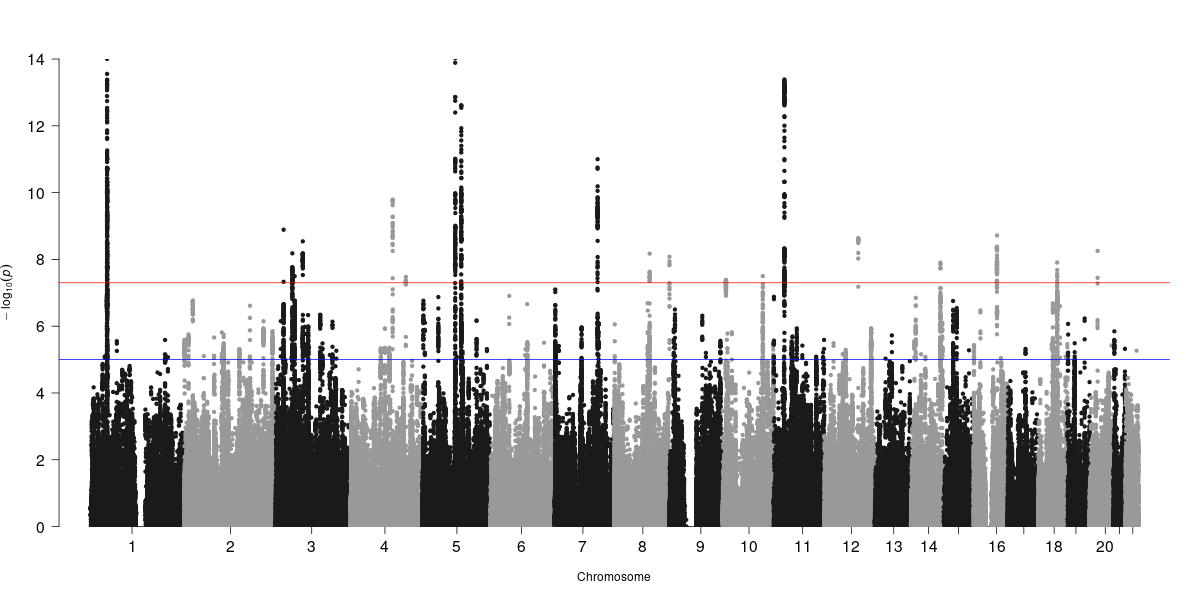


ADHDdiscordant

-log(p-value)

Chromosome


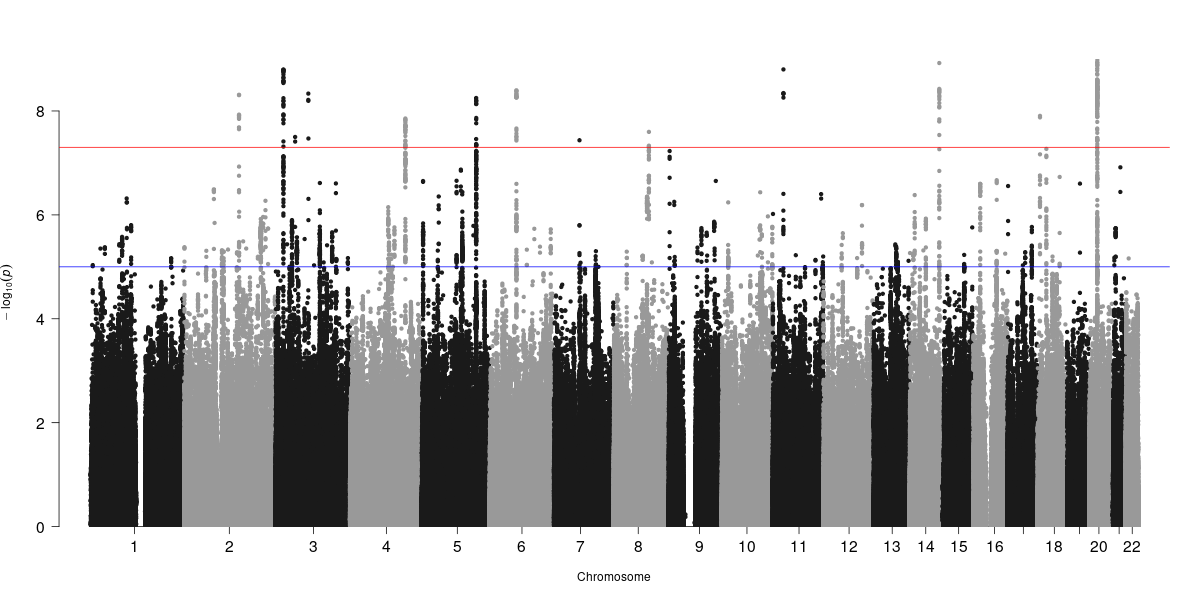


(d)

ASDnoEA

-log(p-value)

Chromosome


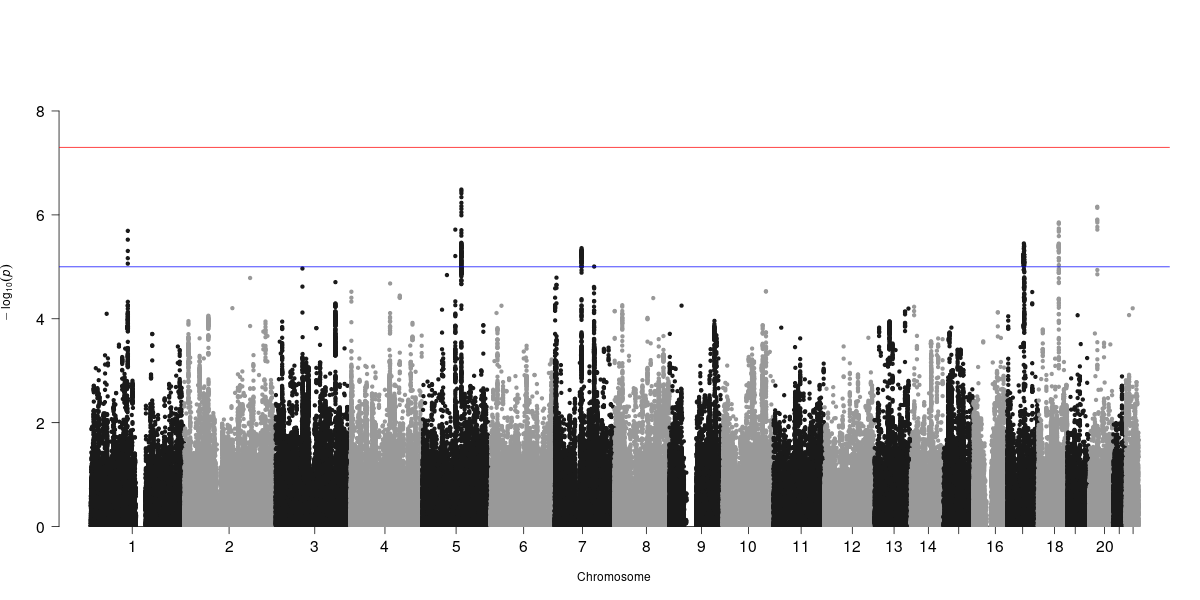


(e)

ASDdiscordant

-log(p-value)

Chromosome


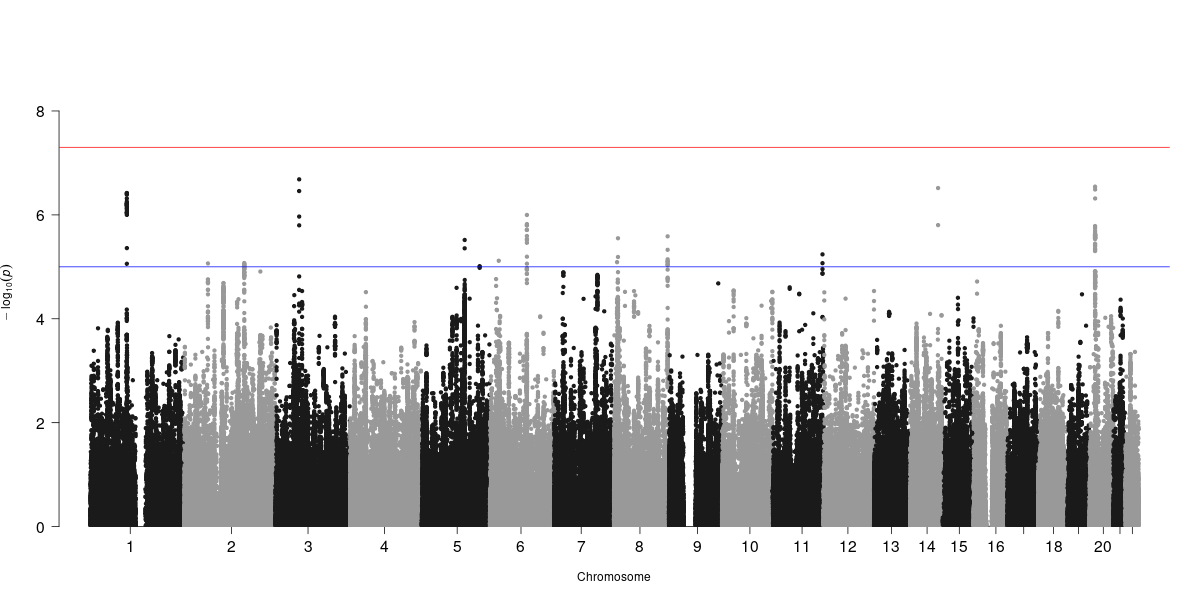


(f)

ASDconcordant

-log(p-value)

Chromosome

**Supplementary Figure 4. Manhattan plots for the ADHD and ASD subsets of variants based on their relationship with EA.** Manhattan plots constructed from the GWAS summary statistics of ADHD ^2^ considering (a) variants not associated with EA (P_EA_>0.05; ADHD_noEA) and (b) variants showing discordant and (c) concordant direction of the effect in ADHD and EA; Manhattan plots constructed from the GWAS summary statistics of ASD ^3^ considering (d) variants not associated with EA (P_EA_>0.05; ASD_noEA) and (e) variants showing discordant and (f) concordant direction of the effect in ASD and EA. Blue line denotes genome-wide suggestive (P<1e-05) and red line denotes genome-wide significance (P < 5E-08).

**Supplementary Figure 5. Results from the partitioned heritability analysis with LDSC using “anti-target” reference annotations.** Plotted bars represent heritability enrichment for 13 brain tissues from GTEx (Finucane et al., 2018) and three brain cell-types (astrocyte, neuron and oligodendrocyte) (Cahoy et al., 2008) across ADHDdiscordant, ADHDnoEA, ASDconcordant, ASDdiscordant and ASDnoEA genomic partitions (ordered from the top to the bottom of the figure). Error bars displayed represent 95% confidence intervals (estimate +/- 1.96*SE). One-sided t-tests were used to evaluate whether the cell-type enrichment within a particular genomic annotation is higher than the associated “anti-target” reference annotation. * Significant results after Benjamini-Hochberg FDR correction (pFDR < 0.05).

(a)

(b)

**Supplementary Figure 6. Annotation-stratified genetic covariance between ADHD or ASD and related neuropsychiatric disorders, cognition and personality traits.** Genetic covariances corrected by sample overlap for four subsets of SNPs: all SNPs, variants not associated with EA and variants associated with EA with concordant and discordant direction of the effect in EA and (a) ADHD or (b) ASD. FDR-corrected significant associations (P_FDR_ < 0.05) are marked with an asterisk.

**BIBLIOGRAPHY**

1. Lee JJ, Wedow R, Okbay A, et al. Gene discovery and polygenic prediction from a genome-wide association study of educational attainment in 1.1 million individuals. *Nat Genet*. 2018;50(8):1112-1121. doi:10.1038/S41588-018-0147-3

2. Demontis D, Walters GB, Athanasiadis G, et al. Genome-wide analyses of ADHD identify 27 risk loci, refine the genetic architecture and implicate several cognitive domains. *Nat Genet*. 2023;55(2):198-208. doi:10.1038/S41588-022-01285-8

3. Grove J, Ripke S, Als TD, et al. Identification of common genetic risk variants for autism spectrum disorder. *Nat Genet*. 2019;51(3):431. doi:10.1038/S41588-019-0344-8

4. Finucane HK, Reshef YA, Anttila V, et al. Heritability enrichment of specifically expressed genes identifies disease-relevant tissues and cell types. *Nature Genetics 2018 50:4*. 2018;50(4):621-629. doi:10.1038/s41588-018-0081-4

5. Cahoy JD, Emery B, Kaushal A, et al. A Transcriptome Database for Astrocytes, Neurons, and Oligodendrocytes: A New Resource for Understanding Brain Development and Function. *The Journal of Neuroscience*. 2008;28(1):264. doi:10.1523/JNEUROSCI.4178-07.2008
